# Adverse health effects of asbestos: solving mysteries regarding asbestos carcinogenicity based on follow-up survey of a Chinese factory

**DOI:** 10.1186/s12199-018-0726-z

**Published:** 2018-08-08

**Authors:** Eiji Yano

**Affiliations:** Teikyo University Graduate School of Public Health, 2-11-1 Kaga, Itabashi-ku, Tokyo, 173-8605 Japan

**Keywords:** Asbestos, Lung cancer, Chrysotile asbestos, Amphibole asbestos, Tremolite, China, Epidemiology, Fiber measurement

## Abstract

The present review summarizes the results of several follow-up studies assessing an asbestos product manufacturing plant in Chongqing, China, and discusses three controversial issues related to the carcinogenicity of asbestos. The first issue is the amphibole hypothesis, which asserts that the carcinogenicity of asbestos is limited to amphiboles, such as crocidolite, but not serpentines, such as chrysotile. However, considering the possible multiple component of asbestos carcinogenicity in the presence of tobacco smoke or other carcinogens, chrysotile cannot be regarded as non-carcinogenic. Additionally, in a practical sense, it is not possible to assume “pure” chrysotile due to its ubiquitous contamination with tremolite, which is a type of amphibole. Thus, as the International Agency for Research on Cancer (IARC) assessed, all forms of asbestos including chrysotile should be regarded carcinogenic to humans (Group 1). The second issue is the chrysotile/tremolite paradox, which is a phenomenon involving predominant levels of tremolite in the lung tissues of individuals who worked in locations with negligible levels of tremolite due to the exclusive use of chrysotile. Four possible mechanisms to explain this paradox have been proposed but this phenomenon does not support the claim that amphibole is inert. The final issue discussed is the textile mystery, i.e., the higher incidence of cancer in asbestos textile plants compared to asbestos mines where the same asbestos was produced and the exposure levels were comparable. This phenomenon was first reported in North America followed by UK and then in the present observations from China. Previously, levels of fiber exposure were calculated using a universal converting coefficient to estimate the mass concentration versus fiber concentration. However, parallel measurements of fiber and mass concentrations in the workplace and exposed air indicated that there are wide variations in the fiber/mass ratio, which unjustifies the universal conversion. It is possible that contamination by airborne non-fibrous particles in mines with mass fiber conversion led to the overestimation of fiber concentrations and resulted in the textile mystery. Although the use and manufacturing of asbestos has been banned in Japan, more than 10 million tons of asbestos had been imported and the majority remains in existing buildings. Thus, efforts to control asbestos exposure should be continued.

## Background

**Fig. 1:**
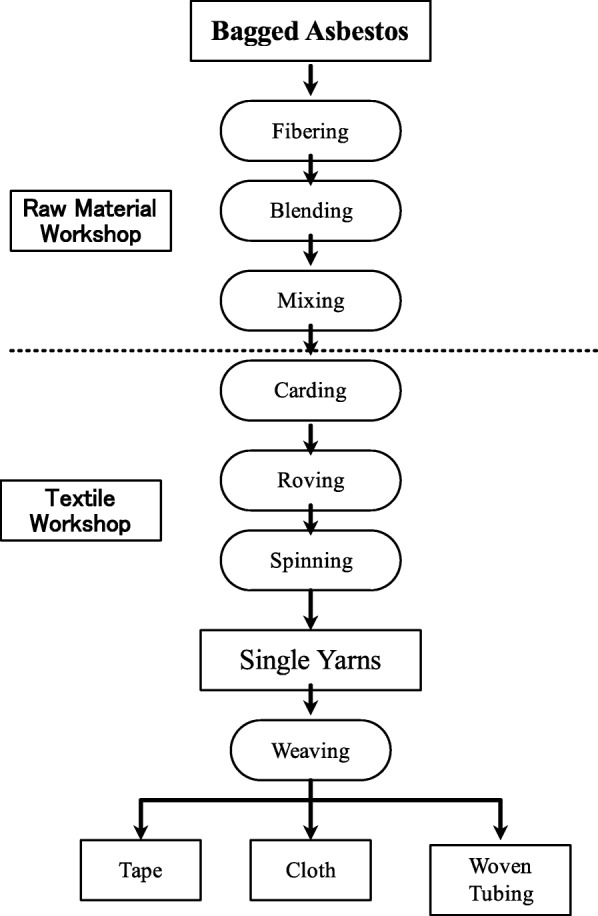
Raw material and textile workshop

**Fig. 2:**
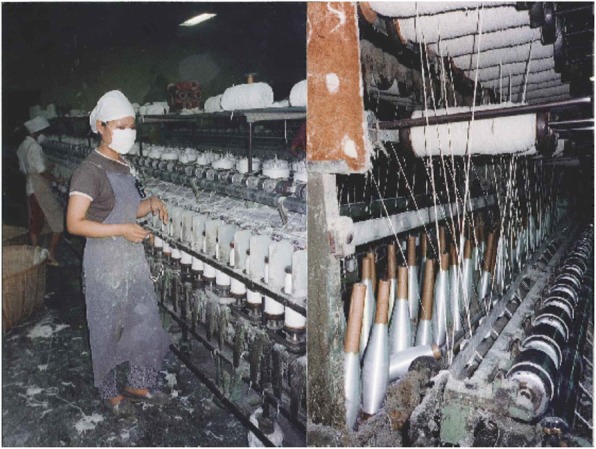
Roving (left) and spinning (right) process

In 1939, the Chongqing asbestos factory began its operation by using water transportation on the Yangzi River to carry in raw materials and ship out manufactured products. This factory contained workshops for raw materials, textiles (carding, spinning, weaving), asbestos cement, rubber, and friction materials (Figs. 1 and 2). Its dust concentration, but not fiber concentration, was measured by Sichuan University (formerly West China University of Medical Sciences) every 5 years. In the 1980s, major innovations to improve the workplace environment included the installation of local ventilation systems throughout the factory, but, beginning in the 1990s, the systems were no longer operated to cut costs. Additionally, a small hut with a scrubber for the final treatment of exhaust air had been turned into a storage area for dumping sacks.

Although the manager of the factory claimed that the only type of asbestos used was chrysotile, he could not totally reject the possibility of contamination with amphibole. Using transmission electron microscopy, Dr. Kohyama, who is an asbestos mineralogy expert, examined samples from two Sichuan asbestos mines whose products were mainly used in the factory and found that the tremolite contamination was less than 0.001% [1]. Later, airborne fiber samples were collected from the workplace and analyzed using scanning electron microscopy; only a small proportion of tremolite was found [2, 3].

At one point, the factory employed approximately 1200 individuals and, as with many Chinese companies, worker residences were on the same premises. Workers went back to their home residence during their lunch break, dined with their families in the evening, and family members often went to the factory to use the bathrooms commonly used by workers. Even after retirement, many workers stayed in the same residence and received a pension from the company. Incredibly, the present authors observed a child playing between factory buildings where suspended asbestos fibers were visible and shining in the sunlight. Thus, although it is beneficial to follow up with workers for epidemiological studies, their family members were also exposed to asbestos at work and in their personal lives throughout their life span and could have been affected as well.

The production of the factory severely decreased during the Cultural Revolution but increased in the late 1980s due to increased exports to Japan and Europe following restrictions that forbade the manufacturing of asbestos products in these countries. During the 1990s, production sharply decreased again, particularly in the cement and textile workshops, and shifted toward information technology-related areas such as electronic circuit cards. Traditionally, in this factory, the retirement age for women was 10 years earlier than that of men and workforce reductions were accomplished mainly by decreasing the number of female workers. The present author has published a number of epidemiological studies on the asbestos-related diseases of workers in this factory over the last two decades [1–16]. Using these data, the present review will summarize important findings obtained from this factory with a focus on several issues that remain controversial in the field, namely, the amphibole hypothesis, the chrysotile/tremolite paradox, and the textile mystery.

### Amphibole hypothesis

Asbestos is a group of fibrous minerals that can be classified into two major groups: amphiboles and serpentines. Amphibole asbestos types include crocidolite, which is known as blue asbestos; amosite, which is known as brown asbestos; and anthophyllite (Table 1).

**Table 1:** Types of asbestos

Serpentine	Chrysotile (white asbestos)
Amphibole	Crocidolite (blue asbestos)
	Amosite (brown asbestos)
	Anthophyllite
	Tremolite
	Actinolite

Although the carcinogenicity of amphiboles, particularly crocidolite, is well recognized, the carcinogenicity of chrysotile, a serpentine known as white asbestos, has long been controversial. Because construction is the largest industry sector where asbestos has been used and a majority of these projects used chrysotile, its carcinogenic potency has huge implications for society. Subsequently, claims have been made that amphiboles, but not serpentines, are carcinogenic [17, 18]; this claim is referred to as the amphibole hypothesis and is supported by the asbestos industry. If this hypothesis stands, asbestos may be used in most cases as long as good standards of control are enforced.

**Fig. 3:**
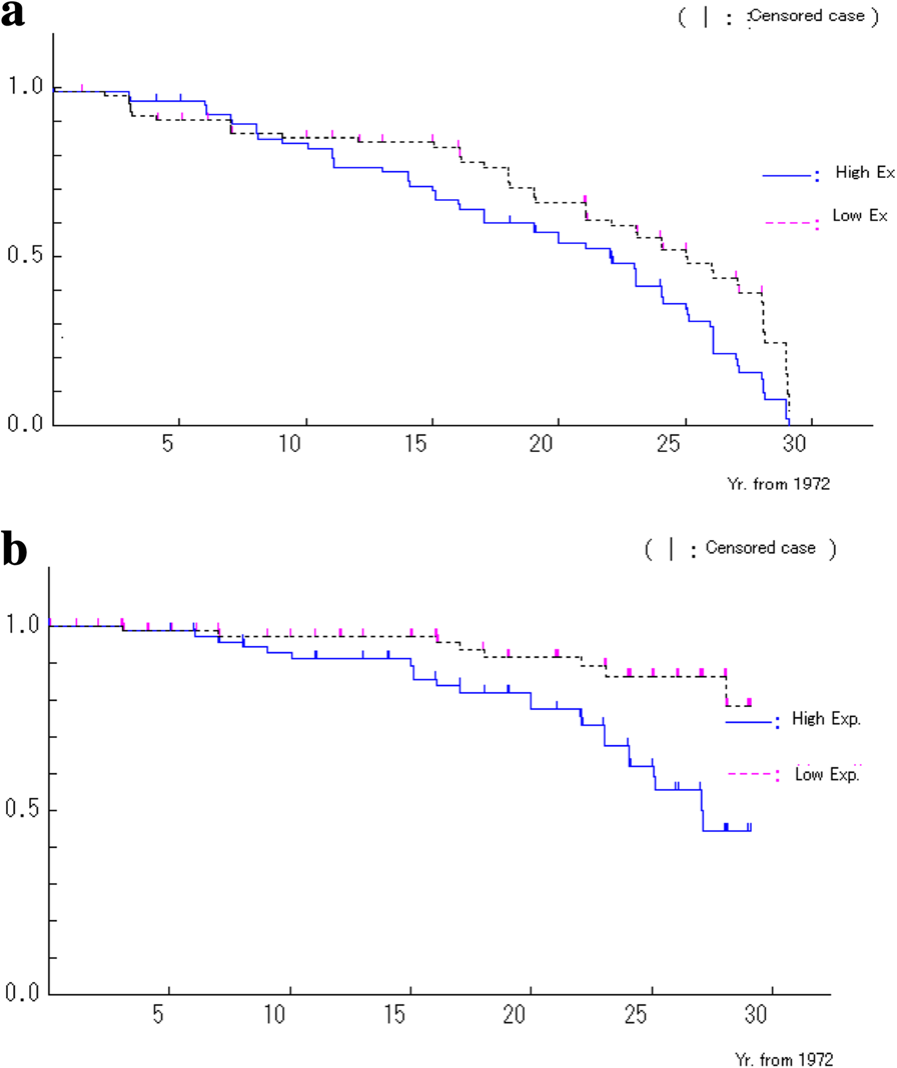
**a** Survival curves of workers in high- and low-exposure workshops. Workshops for raw materials and textile were classified as high-exposure and those of asbestos cement and administration were classified as low-exposure. **b** Survival curves for lung cancer and mesothelioma for workers in high- and low-exposure workshops

In 2001, our research group published a 25-year follow-up investigation of the Chongqing factory in which a cohort of 515 male workers was categorized into high-, medium-, and low-exposure subgroups based on the average level of exposure in each workshop [1]; a proportional hazards model was used to adjust for age and smoking. The rates for adjusted relative mortality (Fig. 3a) and risk of lung cancer (Fig. 3b) were 1.5 (95% confidence interval [CI] 1.0–2.3) and 8.1 (95% CI 1.8–36.1) for workers exposed to high and low levels of asbestos, respectively. These results suggest that heavy exposure to chrysotile asbestos can cause lung cancer in factory workers where amphibole had never been purposefully introduced; this finding was confirmed by several follow-up studies [3–8]. For example, 577 asbestos workers and 435 control workers were followed from 1972 to 2008, and 259 deaths were identified in the asbestos cohort [7]. Of these 259 individuals, the major cause-specific fatalities included 96 deaths from various cancers, such as lung cancer (*n* = 53) and non-malignant respiratory diseases (*n* = 81). In contrast, the control cohort included nine cases of lung cancer and 11 cases of respiratory diseases. The age- and smoking-adjusted hazard ratios for mortality by all causes and all cancers in asbestos workers were 2.05 (95% CI 1.56–2.68) and 1.89 (95% CI 1.25–2.87), respectively, whereas those for lung cancer and respiratory diseases were 3.31 (95% CI 1.60–6.87) and 3.23 (95% CI 1.68–6.22), respectively.

A nested case-control study was conducted with workers from the same factory [8]. Of the 1139 asbestos workers who had ever worked there for longer than 1 year, there were 41 male individuals with lung cancer in 2001 and each case was matched according to age (± 5 years) with five control subjects. A conditional logistic regression model was performed to estimate the odds ratios (ORs) of lung cancer risk associated with different exposure levels and revealed that 54% of cases had high exposure and 24% had low exposure while 24% of controls had high exposure and 44% had low exposure; smoking was more common in the cancer cohort (90%) than in the control group (73%). The adjusted OR for lung cancer was 3.66 (95% CI 1.61–8.29) for the high-exposure group and was slightly elevated for the medium-exposure group (OR 1.25; 95% CI 0.47–3.31); additionally, smoking was related to lung cancer risk (OR 3.33; 95% CI 1.10–10.08). Compared to the low-exposure non-smoking group, the ORs were 10.39 (95% CI 1.34–82.45) for the high-exposure smoking group and 5.23 (95% CI 0.50–54.58) for the high-exposure non-smoking group. These findings indicate that there was a multiplicative interaction between smoking and asbestos for the incidence of lung cancer, which suggests an important role for smoking as a cause of lung cancer in asbestos workers.

Unlike lung cancer (bronchogenic carcinoma), the multiplicative interaction (synergistic effect) between asbestos exposure and cigarette smoking has not been observed for malignant mesothelioma [19]. In the Chongqing factory, there were two confirmed cases of malignant mesothelioma, one was pleural and one was peritoneal, and one suspected case (unconfirmed by histopathology) of plural mesothelioma. The proportion of cases with mesothelioma compared to those with lung cancer in this factory was relatively small compared with other studies [20], which may suggest the important role of smoking in the causal mechanisms of lung cancer among asbestos-exposed populations.

In the Chongqing factory, the prevalence of smoking in asbestos workers was high. If smoking contributes to asbestos-related lung cancer, then it should be included as a consideration in the amphibole hypothesis. It is possible that asbestos causes cancer through multiple mechanisms and the absence of a synergistic effect between asbestos and cigarette smoking in the incidence of mesothelioma suggests that asbestos may cause mesothelioma without other carcinogen or with carcinogens other than cigarette smoking. On the other hand, in relation to lung cancer, asbestos may act with cigarette-derived chemical carcinogens. The production of reactive oxygen metabolites (ROMs), such as hydroxyl radicals, by the interaction between asbestos and phagocytic cells is well recognized, and thus, the mechanisms underlying ROM production induced by various types of asbestos in conjunction with phagocytic cells were investigated [21–23]. Although the potency and mechanism of each type of asbestos may not be the same, both amphibole and serpentine (chrysotile) asbestos types produce ROMs. This can relate to the carcinogenic mechanisms of asbestos, and thus, asbestos of any type can cause cancer.

Recently, our research group published the results of a fiber size analysis of airborne samples collected from each workshop in the Chongqing factory [2]. Scanning electron microscopy was used to measure the lengths and widths of the fibers and identify the fiber types. This fiber type-specific size analysis revealed that tremolite was overrepresented among the total fibers even though it was estimated to be less than 1.5% at its highest concentration. Because tremolite is a natural contaminant of chrysotile and the proportion of tremolite in chrysotile may differ according to feedstock, it is not possible to absolutely avoid contamination with tremolite, i.e., despite industry claims, it is not possible to recognize natural chrysotile as non-carcinogenic due to its unavoidable contamination with amphibole. Taken together with the possible multiple mechanisms of asbestos carcinogenicity, these findings indicate that the amphibole hypothesis, which states that amphibole can cause cancer but chrysotile does not, cannot be accepted. Thus, as the International Agency for Research on Cancer (IARC) assessed, all forms of asbestos including chrysotile should be regarded carcinogenic to humans (Group 1) [24].

### Chrysotile/tremolite paradox

The second controversial issue involves the different asbestos fiber types present in the work environment versus those in the lungs of workers from workplaces exclusively using chrysotile. The vast majority of asbestos in the workplace was chrysotile whereas almost all of the fibers identified in lung tissues were tremolite with only exceptional instances of chrysotile [2]. This odd contrast, which is known as the chrysotile/tremolite paradox [10], has been observed in several studies [25, 26]. For example, McConnochie et al. [25] conducted a fiber type analysis using the lung tissues of patients with mesothelioma and those of local sheep from around a chrysotile mine in Cyprus. A sizable proportion of tremolite that differed from the original composition found in the mine was identified in both the human and sheep lung tissues. A recent report from our research group proposed several possible mechanisms that may explain the paradoxically high levels of tremolite in these lung tissues despite the chrysotile-dominant work environment with only exceptional instances of tremolite [10]. The first explanation relates to the curly shape of the chrysotile fiber that may be more likely to become entrapped in the narrow collider of the airway, which has many bifurcations in the bronchi and bronchioles. In contrast, the straight-shaped tremolite fibers can flow parallel with the air stream and penetrate to the deeper portion of the lung. Furthermore, the aerodynamic properties of the curly-shaped chrysotile fibers may differ from those of the straight-shaped tremolite fibers during the handling process of asbestos in a textile factory. During the raw material process in the factory, preparation of the material by mixing it with vibration and recycling may lead to the selective accumulation of curly chrysotile fibers in the air. Additionally, this may increase the proportion of chrysotile to tremolite in the ambient air from the original material. The accumulation of tremolite in lung tissue may also occur due to its biopersistence whereas chrysotile is rapidly cleared away [27]. Compared with tremolite, chrysotile becomes easily bloated and dissolves in lung fluid, which may result in earlier leaching and clearance from the lung.

Sebastien et al. [26] performed a fiber type analysis according to months of exposure (duration) and months after exposure (cessation) and found that the highest chrysotile content and highest chrysotile/tremolite ratio occurred in the tissues of subjects with the shortest time since cessation; these findings suggest that chrysotile has a rapid clearance rate relative to tremolite. In tissue samples obtained from miners and millers in Quebec, Canada, chrysotile levels had decreased with time since the last exposure whereas those of tremolite did not [25]. In fact, following exposure to chrysotile with minor contamination from tremolite, the number of tremolite fibers in the lung exceeds that of chrysotile 15 years after the cessation of exposure [27–29]. In addition to these biophysical explanations, it is also possible that tremolite concentrations exceed chrysotile concentrations in the lung based on the chemical dissolution of chrysotile fibers in a formalin bath after tissue dissection [30]. Formalin is generally acidic and, following the absorption of carbon dioxide from the air, even neutralized formalin would turn acidic and preferentially dissolve chrysotile to tremolite, which is durable to acid.

The chrysotile/tremolite paradox is sometimes regarded as evidence supporting the amphibole hypothesis. However, for the same reasons mentioned above in conjunction with the likely reasons why chrysotile is less persistent than tremolite in lung tissue, this hypothesis cannot be accepted.

### Textile mystery

The third controversy is the textile mystery, which is derived from the observation that asbestos textile workers in Charleston, South Carolina, had a much greater risk for respiratory cancers than miners and millers from Quebec who had a similar level of exposure to the same source of chrysotile [26]. Similar findings have been reported in studies of workers from Mannheim, Pennsylvania, [31] and Rochdale, UK [32].

To determine if this same phenomenon occurs in China, our group planned a comparative study of a textile factory and a Sichuan chrysotile mine but a team from Japan specifically tasked with fiber monitoring was unable to obtain permission to visit the Chinese asbestos mine where the study was performed. Although this prohibited our group from making fiber measurements using globally accepted standard procedures [33], fiber exposure was estimated by calculating the concentration from dust measurements. This study concurrently observed a chrysotile mining cohort and a chrysotile textile cohort for 26 years. Data regarding vital status, occupational history, and smoking habits were collected, and causes and dates of deaths were verified using death registries. Additionally, individual cumulative fiber exposures were estimated based on periodic dust/fiber measurements from different workshops, job title, and job duration, and the workers were categorized into four levels (Q1–Q4) of exposure. Standardized mortality ratios (SMRs) for lung cancer were calculated and stratified by industry and job title with reference to the national rates. Cox proportional hazard models were fit to estimate the exposure-specific lung cancer risks following adjustments for age and smoking, and an external control cohort consisting of industrial workers without asbestos exposure was used as a reference group for both the textile and mining workers. The SMRs were almost consistent with exposure levels in terms of job title and workshop, but there was a clear exposure-response relationship between lung cancer mortality and exposure levels in both cohorts. At low exposure levels (Q1 and Q2), the textile workers displayed a higher risk of death from lung cancer than the mining workers. However, similarly high risks of death from lung cancer were observed at higher exposure levels (hazard ratios > 8 and 11 at Q3 and Q4, respectively) for both the textile and mining workers after adjusting for both age and smoking. Thus, the textile mystery also occurred in China, at least at relatively low exposure levels.

Sebastien et al. [26] proposed three hypotheses to explain the textile mystery, namely, the possibility of a co-carcinogen in textile plants, the presence of longer fibers in the textile industry, and low reliability of the exposure data. First, different levels of smoking and oil mist with nitrosamine were suspected in the textile factory. Compared with the mining and milling plant, rotating machines were extensively used in the textile plant and the chance for workers to be exposed to machine oil was higher in the latter. Thus, nitrosamine, which is used as a rust inhibitor for machines, was suspected as the culprit. Different levels of smoking were also suspected but no definitive conclusion has been provided as of yet. Second, it is possible that the size distributions of the asbestos fibers differed between the two exposure settings. The mining of chrysotile ore, the crushing and milling of the product, the collection of fibers, and the bagging of the product are the major processes performed in the mine whereas each fiber is extensively processed in a textile plant. This may generate finer fibers and a higher proportion of longer fibers, which could play a role in the fiber type selection suggested by the chrysotile/tremolite paradox.

**Fig. 4:**
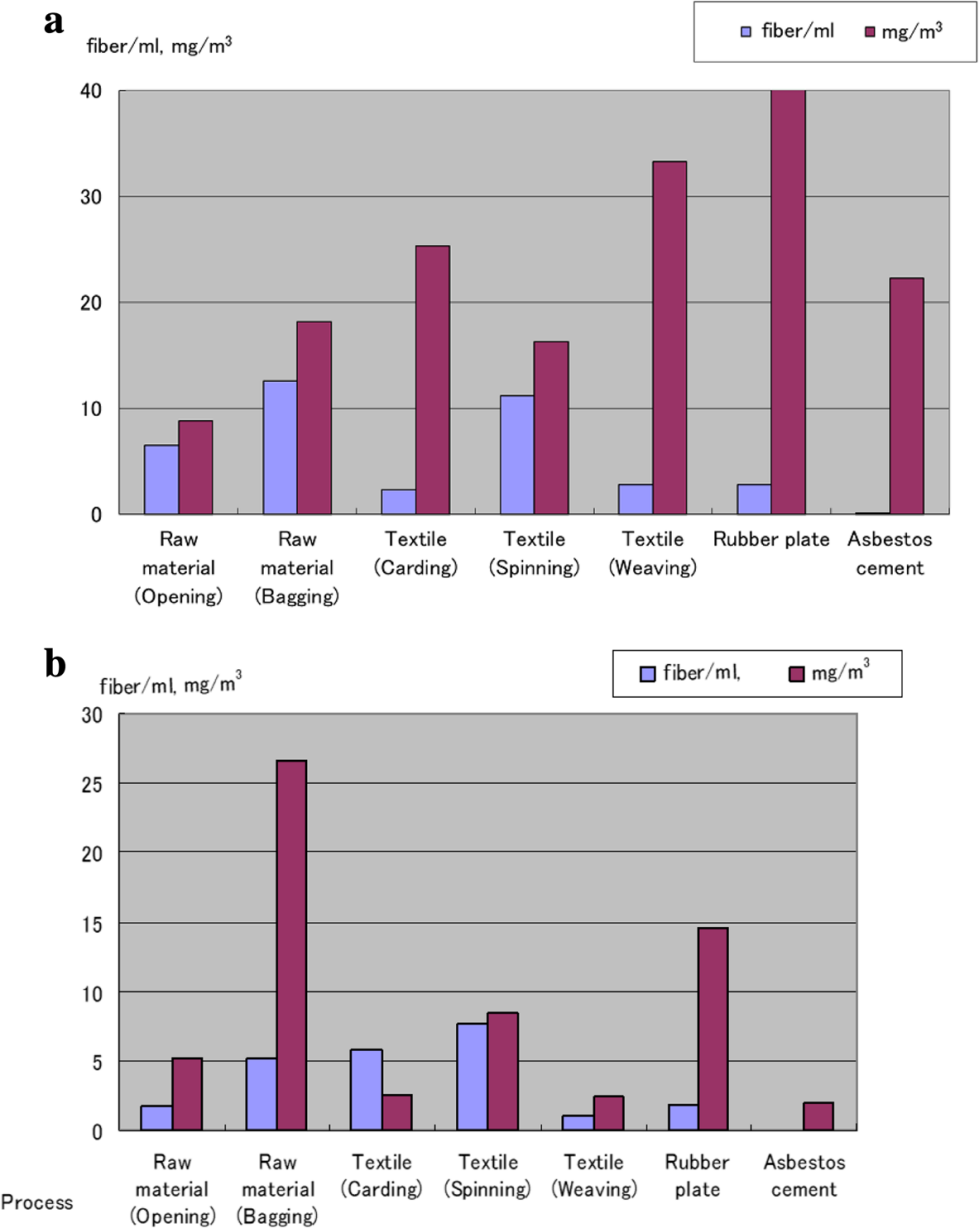
**a** Dust and fiber concentrations obtained by personal sampling. **b** Dust and fiber concentrations obtained by workplace measurements

Although these two explanations are plausible, the third may be the most likely. This assumption implicates the low reliability of exposure data or the overestimation of fiber exposure in the mines due to contaminating dust particles. Previously, exposure to asbestos was almost exclusively estimated by converting the mass concentration (dust weight obtained using the impinger method) into a fiber concentration by applying a single converting factor. However, in the present study, the parallel measurements of mass (mg/m^3^) and fibers (fiber/m^3^) revealed a varying relationship between the dust and fiber concentrations that depended on the workshop (Fig. 4a, b, for detailed methods see [2, 3]). In particular, the relative concentrations of fiber at the asbestos cement and rubber workshops were low compared with the mass concentrations. In the former, the largest component of the dust was cement whereas mica, limestone, rubber, and various materials were mixed with asbestos in the latter. Apparently, it is inappropriate to obtain fiber concentrations from mass concentrations using a single conversion factor. In the mine, the primary job is to separate asbestos fibers from raw rock and purify them, which involves a significant degree of non-fibrous dust, whereas the asbestos fibers used in a textile plant are already purified and in a bag [34]. Thus, the proportion of fiber in the dust at a mine would be lower than at a textile plant and would lead to an overestimation of the fiber concentration at the mine if the same conversion coefficient was used for both workplaces. A comparison of the same levels of dust exposure between the mine and textile plant would result in a much higher fiber exposure at the textile plant, and thus, it is understandable that there would be more cases of lung cancer in this type of workplace.

Despite strenuous efforts to confirm speculation that the textile mystery is due to the overestimation of fiber concentrations in the mine, it is not possible to accomplish this in the absence of exact measurements of fiber concentrations. Thus, the speculation that this can explain the textile mystery shall remain.

## Conclusions

The present review addressed three controversial issues regarding the understanding of epidemiological findings related to asbestos based on observations from the asbestos industry in China. Based on these findings, it is possible to reject the amphibole hypothesis and its implication that all asbestos, regardless of fiber type, should be banned from industrial use and eliminated from the environment. Although the use and manufacturing of asbestos have been banned in Japan, more than 10 million tons of asbestos had been imported. As a result, it is important to consider human exposure to asbestos in old buildings and waste dumping from construction sites due to the persistence of asbestos in the environment and to continue efforts to control asbestos and its use.

## Abbreviations

CI: Confidence interval
OR: Odds ratio
